# Regulation of cGAS‐Mediated Immune Responses and Immunotherapy

**DOI:** 10.1002/advs.201902599

**Published:** 2020-02-06

**Authors:** Abdullah F. U. H. Saeed, Xinglin Ruan, Hongxin Guan, Jingqian Su, Songying Ouyang

**Affiliations:** ^1^ The Key Laboratory of Innate Immune Biology of Fujian Province Provincial University Key Laboratory of Cellular Stress Response and Metabolic Regulation Biomedical Research Center of South China Key Laboratory of Optoelectronic Science and Technology for Medicine of Ministry of Education College of Life Sciences Fujian Normal University Fuzhou 350117 China; ^2^ Fujian Key Laboratory of Special Marine Bio‐resources Sustainable Utilization The Public Service Platform for Industrialization Development Technology of Marine Biological Medicine and Product of State Oceanic Administration College of Life Sciences Fujian Normal University Fuzhou 350117 China; ^3^ Laboratory for Marine Biology and Biotechnology Pilot National Laboratory for Marine Science and Technology (Qingdao) Qingdao 266237 China; ^4^ College of Chemistry and Materials Science Fujian Normal University Fuzhou 350117 China; ^5^ Department of Neurology Fujian Medical University Union Hospital 29 Xinquan Road Gulou District Fuzhou 350001 China

**Keywords:** cGAS‐STING, cytosolic sensing, immunotherapy, innate immune regulation, tumorigenesis

## Abstract

Early detection of infectious nucleic acids released from invading pathogens by the innate immune system is critical for immune defense. Detection of these nucleic acids by host immune sensors and regulation of DNA sensing pathways have been significant interests in the past years. Here, current understandings of evolutionarily conserved DNA sensing cyclic GMP‐AMP (cGAMP) synthase (cGAS) are highlighted. Precise activation and tight regulation of cGAS are vital in appropriate innate immune responses, senescence, tumorigenesis and immunotherapy, and autoimmunity. Hence, substantial insights into cytosolic DNA sensing and immunotherapy of indispensable cytosolic sensors have been detailed to extend limited knowledge available thus far. This Review offers a critical, in‐depth understanding of cGAS regulation, cytosolic DNA sensing, and currently established therapeutic approaches of essential cytosolic immune agents for improved human health.

## Graphical abstract

The cGAS‐STING pathway is central to innate immunity and must be tightly regulated. cGAS performs essential roles in numerous immune functions such as DNA sensing, antiviral defense, antitumor immunity, senescence, and DNA repair. Aberrant cGAS activation leads to autoimmune and inflammatory diseases. Hence, several therapeutic approaches are used to regulate cGAS and related cytosolic sensors for improved human health.

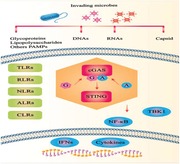

## 1 Introduction

**Figure 1:**
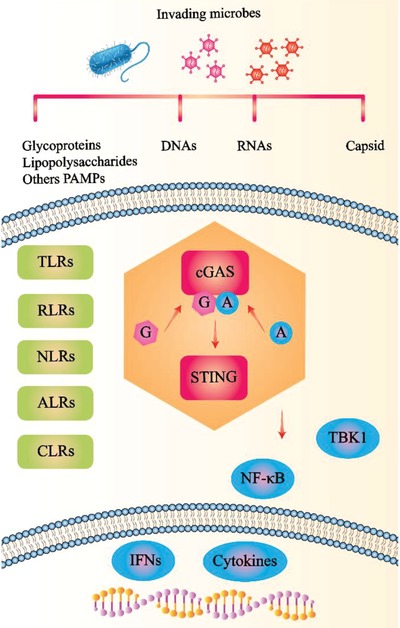
PRRs recognized PAMPs, evolutionarily conserved features derived from bacteria, fungi, parasites, and viruses, to avert pathogen invasion. PAMPs from invading microbes activate PRRs, including TLRs, RLRs, NLRs, and CLRs. Subsequently, PRRs trigger cGAS‐STING immune pathways, which lead to the induction of IFNs and pro‐inflammatory cytokines. PRRs: pattern‐recognition receptors; RLRs: RIG‐I‐like receptors; NLRs: nucleotide oligomerization and binding domain (NOD)‐like receptors; ALRs: AIM2‐like receptors; CLRs: C‐type lectin‐like receptors; PAMPs: pathogen‐associated molecular patterns.

The innate immune system, armed with germline‐encoded receptors called pattern‐recognition receptors (PRRs), is on the front line of defense to recognize infectious pathogen‐associated molecular patterns (PAMPs) of disease‐causing pathogens.^1^ PRRs include toll‐like receptors (TLRs), retinoic acid‐inducible gene‐I‐like receptors (RLRs), NOD‐like receptors (NLRs), C‐type lectin‐like receptors (CLRs) **Figure** **1**), and several other nucleic acid receptors.^2^ For more than a decade, there have been remarkable developments in comprehending the signaling mechanisms of innate immune pathways. Studies have confirmed the retinoic acid‐inducible gene I (RIG‐I)/melanoma differentiation‐associated gene 5 (MDA5)–mitochondrial antiviral‐signaling protein (MAVS) axis and the cGAS–stimulator of interferon genes (STING) axis as key nucleic acid recognition pathways. Nevertheless, the proper function of immunostimulatory exogenous nucleic acids in cytosolic sensing remains unclear.^3^

In addition, aberrant detection of self‐nucleic acids, mainly double‐stranded deoxyribose nucleic acids (dsDNAs), can predict the outcome in devastating illnesses.^4^ Besides, the overactivation of this critical immune pathway contributes to the outcome in autoinflammation and autoimmune disease progression.^5^ cGAS‐STING‐mediated antiviral cellular response initiates downstream signaling pathways, which stimulate TANK binding kinase 1 [TBK1, an IKK (IκB kinase)‐related kinase]. Subsequently, TBK1 plays a significant role in regulating innate immunity and activating type I interferon (IFN) regulatory factor 3 (IRF3).^6^ IRF3 is essential for the transcription of immune responsive genes, comprising IFN, and immune‐modulatory cytokines.^3^ The products of these genes cooperatively suppress the proliferation of a broad range of viral entities, such as herpes simplex virus type 1 (HSV1), Kaposi's sarcoma‐associated herpesvirus (KSHV), hepatitis C virus (HCV), and Murine gammaherpesvirus 68 (MHV68).^1^, ^7^, ^8^

cGAS (likewise identified as C6ORF150 and Mab‐21 domain having 1, MB21D1) recognizes cytosolic dsDNA and activates assembly of the second messenger, cGAMP, to activate STING (correspondingly known as MITA, ERIS, MPYS, and TMEM173).^9^ Cytosolic DNA can originate from numerous sources, including viruses, bacteria, fungi, parasites, damaged cells, and DNA‐containing cellular organelles, as well as cancer/tumor cells.^3^ cGAS‐STING‐mediated pathways are strictly regulated to ensure balanced immune responses.^10^ Additionally, the viruses above encode multiple cGAS‐STING antagonists and exploit diverse strategies to evade host antiviral immunity and cause infectious diseases and cancers. Therefore, recognition of the approaches that viral proteins employ to escape cGAS and STING is beneficial for the development of novel therapeutic drugs.^11^ Moreover, cGAS is essential for senescence.^12^ Naturally occurring cellular senescence barricades induction of tumorigenesis and adds to the advancement of antitumor responses of numerous therapies, consisting of radiation and chemotherapy. Similarly, cGAS shows significant regulatory functions in tissue repair, fibrosis, and aging.^12^

By recognizing pathogen‐derived biochemical signatures, consisting of nitrogen bases, lipids, proteins, and sugar and its mixes, innate cytosolic sensors contribute crucial functions in primary innate immune responses.^13^ Many ribonucleic acid (RNA) cytosolic sensors were defined in earlier years, including various RLRs, such as RIG‐I, MDA5, and laboratory of genetics and physiology 2 (LGP2). Additionally, NOD‐, LRR‐ and pyrin domain‐containing protein 3 (NLRP3) is another cytosolic sensor that detects cytosolic dsRNA and bacterial RNA and augments the maturation of interleukin (IL)‐1β and IL‐18 through the instigation of caspase‐1 for antiviral and inflammatory immune responses.^14^ Numerous other cytosolic sensors function in recognition of cytosolic RNA. Protein kinase R (PKR) detects endogenous dsRNAs associated with nuclear and mitochondrial signals, regulates nuclear factor (NF‐κB) pathways, and induces the expression of NLRP3.^15^ Further, IFN‐induced protein with tetratricopeptide repeats (IFIT) family members sense cytosolic RNA and are promptly induced through infection by IFN‐dependent and ‐independent signaling pathways.^16^ Nucleotide‐binding oligomerization domain 2 (NOD2) is identified as a viral PRR that can sense viral ssRNA genomes by interacting with MAVS, which results in the activation of IRF3 to trigger IFN production and antiviral defense.^17^ A new study revealed a novel sensor, known as nuclear matrix protein scaffold attachment factor A (SAF‐A; also known as heterogeneous nuclear ribonucleoprotein U [HnRNPU]), which is a nuclear viral dsRNA sensor for both DNA and RNA viruses.^18^

Several cytosolic DNA sensors are known for antiviral immune responses. IFN‐inducible protein Z‐DNA binding protein 1 (ZBP1; also named as DNA‐dependent activator of IFN regulatory factors [DAI] and DLM‐1) detects cytosolic microbial DNA and functions in host defense responses. LRR binding FLII interacting protein 1 (LRRFIP1) recruits and induces β‐catenin, resulting in IRF3‐dependent production of IFN.^19^ The DEAD‐box helicase 41 (DDX41) sensor, a member of the DEAD‐box proteins, recognizes cytosolic DNA and binds with STING to activate TBK1 and downstream signaling for IFN production.^20^ Recently, Ku heterodimers (Ku70 and Ku80) were identified as DNA‐binding proteins. Ku70 works as a cytosolic PRR recognizing DNA and triggers the production of IFN‐λ1 (type‐III IFN) through the initiation of IFN regulatory factor (IRF)‐1 and IRF‐7.^21^ Also, meiotic recombination 11 homolog A (MRE11) is required for intracellular dsDNA responses, STING trafficking, and IFN induction.^2^ DNA repair is critical in innate immunity. The DNA‐dependent protein kinase (DNA‐PK) cytosolic sensor functions in DNA double‐strand break (DSB) repair by regulating breaks by autophosphorylations in binary collections of sites (ABCDE and PQR), V(D) J recombination events, and p53‐dependent apoptotic response in cells with considerably shortened telomeres.^22^

**Figure 2:**
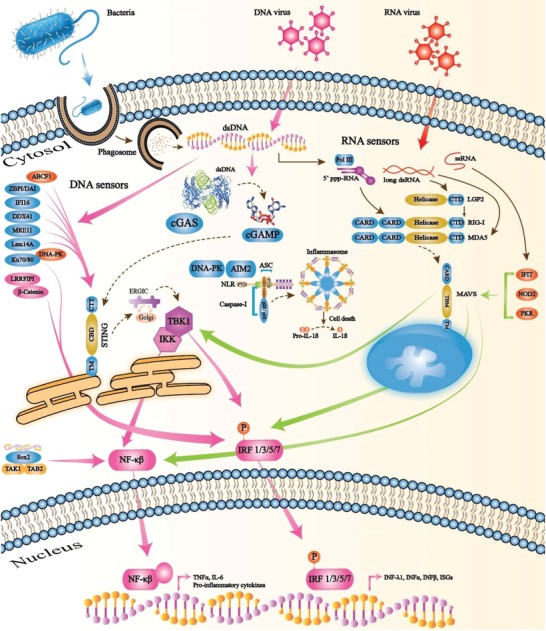
Cytosolic nucleic acid sensors and recognition of innate immune pathways. Nucleic acids (i.e., ssRNA, dsRNA, and DNA) presented by viruses, bacteria, and impaired host cells are leaked and recognized by DNA sensors in the cytosol. During infection, foreign nucleic acids are recognized by RLRs, non‐RLRs, and cGAS, which lead to the induction of IFNs by adaptor proteins MAVS and STING, and transcription factors NF‐κB, IRF1, IRF3, IRF5, and IRF7. Pol III, polymerase III; LGP2, laboratory of genetics and physiology 2; RIG‐I, retinoic acid‐inducible gene I; MDA5, melanoma differentiation‐associated protein 5; IFIT, IFN‐induced protein with tetratricopeptide repeats; NOD2, nucleotide‐binding oligomerization domain 2; PKR, protein kinase R; AIM2, absent in melanoma 2; DNA‐PK, DNA‐dependent protein kinase; cGAS, cyclic GMP‐AMP synthase; ZBPI/DAI, Z‐DNA binding protein 1/DNA‐dependent activator of IFN regulatory factors; IFI16, IFN‐gamma inducible protein 16; MRE11, meiotic recombination 11 homolog A; Lsm14A, LSM14A mRNA processing body assembly factor; Ku70/80, Ku heterodimer; LRRFIP1, LRR binding FLII interacting protein 1; DDX41, DExD/H‐box helicase.

Sensing cytosolic pathogens and cellular perturbations are exceedingly vital. AIM2‐like receptor (AIM2) cytosolic sensor recognizes cellular DNA and initiates the assembly of multiprotein complexes named inflammasomes (acute regulators of intestinal tissue) to govern caspase‐1 and caspase‐4/5 (caspase 11 in mice), necessary for the maturation of IL‐1β and IL‐18.^23^ Another intracellular microbial cytosolic DNA sensor for the induction of IFN‐beta (IFN‐β) is IFN‐γ‐inducible factor 16 (IFI16).^24^ IFI16 is demonstrated to function in DNA‐driven IFN responses and is related to stimulation of IFN‐alpha (IFN‐α) and IFN‐β,^25^ and aids DNA recognition by cGAS,^25^ in addition to promoting DNA‐driven STING‐dependent signaling.^26^ Likewise, DNA‐dependent RNA polymerase III (Pol III) senses cytosolic DNA and produces RNA, through detection of the subsequent RNA by RIG‐I and the instigation of the downstream signaling pathways.^27^ Furthermore, a sequence‐specific DNA sensor known as Sox2 directly recognizes cytosolic DNA with its high‐mobility‐group (HMG) domain. Sox2 triggers the transforming growth factor beta‐activated kinase 1 (TAK1) and its interacting partner TGF‐beta activated kinase 1 (MAP3K7) binding protein 2 (TAB2), thereby activating the transcription factor NF‐κB for innate immunity.^1^ These cytosolic sensors and their innate immune pathways have become an immunotherapy target for the treatment of infectious diseases (**Figure** **2**).^28^ Sensing microbial signatures triggers signaling pathways resulting in the initiation of transcription factors, comprising NF‐κB and IRFs, inducing the production of IFNs, including pro‐inflammatory cytokines.^29^

Innate immune sensors play a vital role in the early sensing of infectious DNA. However, many questions remain concerning the detailed regulation of cGAS‐mediated innate immunity and the impact on cancer immunotherapy. Therefore, to understand the correct functioning of cGAS in immune responses, we detail its regulation and function regarding immune pathways, as well as its therapeutic role in antitumor responses.

## 2 Structural Biology and Biochemistry of cGAS

Evolutionarily conserved recognition of cytosolic DNA of microbial origin is critical to launching a defense in response to contagious diseases. This recognition mechanism allows the host to differentiate between extraneous DNA and self‐DNA. cGAS produces endogenous second messenger cGAMP from adenosine triphosphate (ATP) and guanosine triphosphate (GTP) in the occurrence of DNA. cGAMP, basically parallel to cyclic dimeric guanosine monophosphate (c‐di‐GMP) and cyclic dimeric adenosine monophosphate (c‐di‐AMP), interacts and initiates closed conformation of cytosolic STING, with an affinity of ≈10 nm, significant for downstream signaling and stimulation of IFN pathways.^30^

cGAS comprises 522 amino acid residues, where the N‐terminus contains about 160 residues. The positive charged *N*‐terminal domain of cGAS enhances its function and plays critical regulatory roles in binding to dsDNA, the formation of lipid droplets promoting phase separation, production of cGAMP, and the threshold for dsDNA sensing by determining the length of dsDNA molecules.^31^ Additionally, ligand‐mediated allostery places cGAS in a standby position, anticipating adjustments to the signaling pathway in a switch‐like fashion.^32^ cGAS holds an amazing structural resemblance to the antiviral cytosolic dsRNA sensor 2′‐5′oligoadenylate synthase (OAS1), nonetheless comprises distinctive zinc (Zn) thumb that identifies B‐form double‐stranded DNA. Crystal structure details of the nucleotidyltransferase domain of cGAS demonstrate the role of DNA sensor in a sequence‐independent mode.^33^

**Figure 3:**
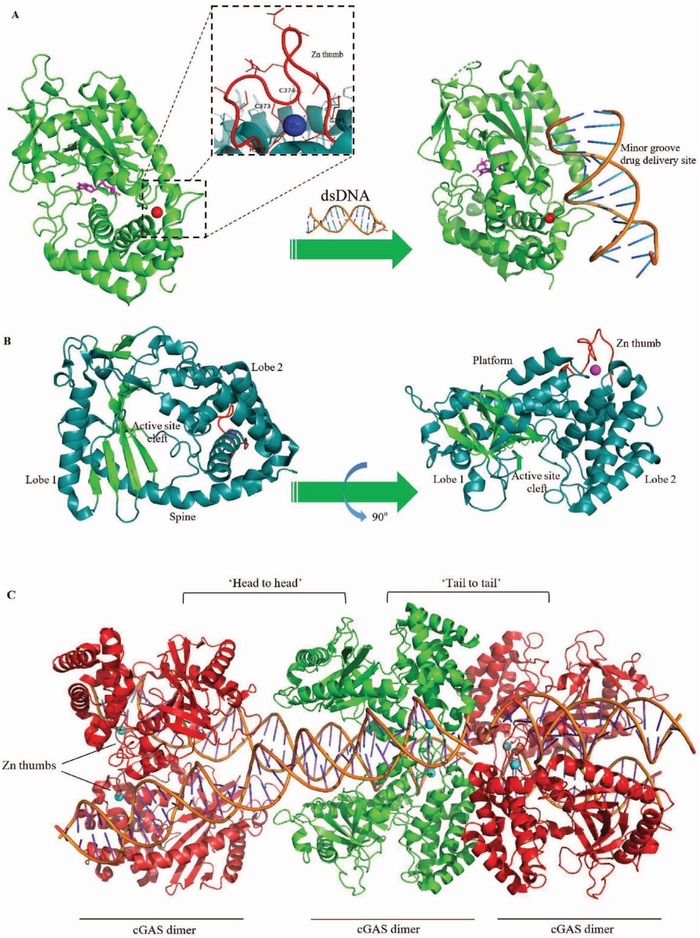
cGAS activation structure and orientation in cGAS‐DNA dimer complex. A) cGAS exists in the apo form in auto‐inhibited conformation (PDB code 4KB6), and detailed observation of the “zinc‐thumb.” Binding to the sugar‐phosphate spine of DNA results in the exposure of cGAS‐DNA composites and cGAS‐active catalytic sites by structural rearrangements for nucleotide binding and catalysis. DNA minor groove is the target drug delivery site employed for therapeutics. B) Ribbon representation of the side views of the cGAS model with marked domains and structures. (cyan α‐helices, green β‐strands; PDB code 4JLX). C) cGAS dimers engage DNA along with zinc (Zn^2+^)‐thumb dimerization elements (PDB code 5N6I). The interchanging “head‐to‐head” or “tail‐to‐tail” assemblage leads to ladder‐like cGAS association over quasi‐continuous DNA in the crystal lattice.

Initial structural and biochemical investigations showed the basic mechanism of enzyme activation and 2′3′‐cGAMP, and relied primarily on mouse cGAS and additional mammalian cGAS homologs that display improved activity and in vitro stability.[qv: 33a] Human cGAS structures exist as a monomer in the inactive form. Its apo form signifies the auto‐inhibited conformation, as well as 2′3′‐cGAMP bound form and sulfate bound form, cGAS has a conserved triggered loop that is positioned adjoining the primary DNA binding surface, and upon DNA binding for biochemical activation, shows switch‐like conformational modifications. cGAS forms a 2:2 complex, which comprises dimeric cGAS, which interacts with two DNA molecules. It binds DNA predominantly by sequence‐independent contacts in cooperation with phosphate‐sugar backbone strands beside the minor groove (**Figure** **3**A,B).[qv: 33a] Similarly, biochemical and structural information propose that the regulation of human‐specific cGAS controls enzyme triggering by biasing cGAS–DNA contacts away from a marginal 2:2 complex and in the direction of higher‐order protein–DNA oligomerization.[qv: 33b] Moreover, the twofold DNA binding planes along with the protein–protein edge of cGAS are vital for activating IRF3, IFN‐β induction, and target therapy for effective drug delivery.[qv: 33a] Exclusively, DNA interacts with Zn thumb and spine. This interaction is crucial for the initiation of cGAS enzyme, and zinc‐ribbon covering exceedingly conserved positively charged amino acids are indispensable for DNA recognition.^34^ The two cGAS dimers are organized in a “head‐to‐head” alignment beside the DNA. Surprisingly, this cGAS_4_–DNA_2_ complexes additionally form a DNA–protein ladder with alternate “head‐to‐head”‐ and “tail‐to‐tail”‐aligned cGAS dimers (Figure 3C). The DNA is sandwiched among “head‐to‐head”‐aligned cGAS dimers and quasi‐continuous (stacked 3′ to 3′ and 5′ to 5′) between the “tail‐to‐tail”‐aligned cGAS dimers. Accordingly, the two dimer interfaces and the DNA binding surface are vital for DNA binding.^34^

cGAS manufactures a cGAMP isomer that strongly interacts with STING and induces a robust IFN response. The endogenous cGAMP produced by cGAS possesses a phosphodiester linkage amid the 2′‐OH of GMP and the 5′‐phosphate of AMP and additionally flanked by 3′‐OH of AMP and the 5′‐phosphate of GMP. Subsequently, the explicit isomer of cGAMP with 2′‐5′, 3′‐5′ linkages is named 2′3′‐cGAMP and is recognized after customary cGAMP (with 3′‐5′, 3′‐5′ linkages, and named 3′3′‐cGAMP) and additional cyclic dinucleotides (CDNs), (for example, c‐di‐AMP and c‐di‐GMP) released from invading microbes.^35^

Following, 2′3′‐cGAMP functions via a subsequent activator that binds to STING, an endoplasmic reticulum (ER)‐membrane adaptor, and induces a conformational modification prompting STING activation. After that, STING translocates from the ER to the Golgi. During this process, the carboxyl end of STING interacts with TBK1 and promotes phosphorylation and dimerization of IRF3.^36^ STING triggers IKK, which then phosphorylates kappa B alpha (IκBα) inhibitor, resulting in its degradation by the ubiquitin‐proteasome pathway, ultimately releasing NF‐kB to the nucleus. STING also phosphorylates and activates IRF3, which, together with NF‐kB, promotes transcription of IFNs and tumor necrosis factor (TNF), IL‐1β, and IL‐6 inflammatory cytokines.^9^

## 3 Activation and Regulation of cGAS‐Mediated Cytosolic DNA Sensing

Although the control of cGAS‐mediated immune responses remains to be investigated, considering the associated processes may shed light on the systems of innate immunity and autoinflammatory ailments, and offer potential therapeutics for drug mediation.^37^

Due to lack of sequence specificity, cGAS can recognize diverse DNA forms, together with self‐DNA,^38^ ssDNA, short dsDNA (≈15 base pairs in length) in vitro, extended DNA lengths in vivo, guanosine (G)‐ended Y‐form short DNA (G‐YSD),^39^ and oxidized DNA.^40^ Recently, it has been shown that Mn^2+^ drives cGAS enzymatic action and sensitivity to dsDNA. It also increases the affinity of adaptor STING to bind with the cGAMP ligand.^1^ In contrast, the mechanism of cGAS dormancy in cells is as yet unknown. However, ongoing studies have proven that genomic DNA harm or autophagy, cytosolic chromatin fragments (CCFs),^41^ micronuclei, chromosomal instability,^42^ and self‐DNA escape could lead to pathophysiological outcomes, resulting in inflammatory reactions initiated by cGAS. DNA damage and genomic instability activate cGAS, which links DNA damage to inflammation, cancer, and cellular senescence.^43^ A new study proposes that calcium and related calmodulin‐mediated signaling regulates cGAS‐STING together with autoimmunity via stimulatory and inhibitory mechanisms. The changes in calcium flux that follow STING activation regulates autophagy for the clearance of intracellular pathogens.^44^ In addition, a CCHC‐type zinc‐finger protein (ZCCHC3) was recently identified as a progressive regulator of cytosolic dsDNA‐ and DNA virus‐induced innate signaling. It has been shown that ZCCHC3 openly interacts with dsDNA, augments the binding of dsDNA to cGAS, and is crucial for cGAS stimulation during infectious diseases.^2^ Another study revealed that cGAS drives non‐canonical inflammasome initiation in age‐related macular degeneration. Additionally, cGAS has shown cGAS‐driven IFN signaling as a channel intended for mitochondrial damage‐triggered inflammasome.^45^ Viral proteins play crucial roles in the regulation of cGAS cytosolic sensing. Zika virus (ZIKV) infection prompts NLRP3 inflammasome induction, which is further improved by viral nonstructural protein (NS) 1 to aid replication. ZIKV triggers NLRP3 activation and regulates cGAS cleavage through NS1. NLRP3 deficiency promotes IFN assembly and reinforces host resistance to ZIKV in vitro and in vivo. Thus, modifying the interaction between inflammasome and IFN signaling may lead to the development of potential therapeutics.^46^

## 4 Regulation of Innate Immune Responses

Host immunity is strictly regulated through several strategies, comprising posttranslational modifications (PTMs), host elements, as well as viral proteins. Other than evading recognition through evolutionary alterations of microbial signatures, pathogens are capable of producing various compounds that interfere with the host defense.^47^ These tactics include sensor downregulation, hindrance of signal transduction innate immune pathways, and disrupting translation. Several host elements regulate intracellular pathogenic nucleic acids. For example, cytosolic self or non‐self DNAs are regulated by exo‐ and endo‐nucleases SAM domain and HD domain‐containing protein 1 (SAMHD1), three prime repair exonuclease 1 (TREX1), deoxyribonuclease II (DNase II), and ribonuclease H2 (RNase H2),^48^ while the viral capsid is ubiquitinated for proteasomal degradation.^49^

### 4.1 cGAS‐Mediated Immune Regulation by PTMs

cGAS is potentially subjected to PTMs, which are essential for host immune regulation (**Table** **1**).^50^ The kinase akt26 activity is inhibited via phosphorylation at Ser305 (Ser291 in mouse cGAS). Akt kinase shows inhibitory effects in cGAS‐mediated antiviral immunity. Ser305 is positioned at the entry of the active site; its phosphorylation generates a negatively charged phosphate group that sterically blocks access to ATP and GTP,^51^ leading to suppression of enzymatic activity, reduced cGAMP production, and IFN‐β production. As a result, phosphorylation of cGAS at this site leads to elevated HSV1 titers post‐infection.^52^

**Table 1:** Regulation of cGAS‐mediated innate immune responses by posttranslational modifications

	Regulatory mechanism	Regulatory function	Regulatory effect	Prospective problem	Reference
Post‐translational modification (PTM)	Phosphorylation	Akt protein phosphorylation at Ser305 or Ser291 sites of cGAS inhibits its catalytic activity	Impaired cGAMP synthesis, and IFNs	How to reverse the inhibition of cGAS‐mediated signaling by phosphatase?	59
		cGAS is phosphorylated at Ser305	Inhibits cGAMP‐synthesis	The activity of cGAS in anti‐tumor immunity remains poorly understood	58
	Ubiquitination	TRIM56 triggers the cGAS‐Lys335 monoubiquitination	Improves dimerization of cGAS, DNA‐binding action, and cGAMP synthesis	In what way TRIM56‐mediated monoubiquitination upsets cGAS dimerization and DNA‐binding activity?	60
		E3 ligase RNF185 catalyzes the ubiquitination of cGAS	Enhance production of IFNs	By what means K27‐linked ubiquitination of cGAS and enzymatic response is modulated?	57
		K48‐linked ubiquitination of cGAS	Impairs IFNs production	E3 ubiquitin ligase accountable this practice is unidentified	3
	Glutamylation	Glutamylation of cGAS by TTLL4 and TTLL6	TTLL6 dampens DNA binding activity, and TTLL4 blocks the synthase activity of cGAS	How do these enzymes function to regulate cGAS activity?	62
	SUMOylation	TRIM38 prevents cGAS for K48‐linked ubiquitination and degradation	Ensures regulation and triggering of the cGAS‐STING immune pathway	Optimal stimulation and shutting of cGAS‐STING immune pathway remains unclear; function of Senp2 at the advanced phase of viral contagion remains unclear	1
		SENP7 protease deSUMOylates cGAS	Activates SUMOylated cGAS	Distinct mechanistic function of SUMOylation in cGAS‐dsDNA cytosolic sensing response remains unclear	64
Cross talk	Autophagy	Beclin‐1 autophagy protein interacts with cGAS	Impairs cGAS, decreases cGAMP synthesis and impairs IFNs	Probably IFI16, DDX41, or additional cytosolic DNA sensors likewise aim Beclin‐1 and prompt autophagy?	65
		TRIM14 inhibits autophagic degradation of cGAS	Inhibits degradation of cGAS and enhance the production of IFN	Distinct regulation of cGAS by ubiquitination remains to be elucidated	3
	Inflammasome	Caspase‐1 interacts and cleaves cGAS	Impedes cGAMP production and IFN induction	Molecular basis of caspases in balancing between IFN and inflammasomes remain unclear; caspase inhibitors should be closely investigated in trials and for antiviral drugs	66

Protein ubiquitination is an essential PTM, which regulates several cellular processes.^58^, ^67^ Several ubiquitin E3 enzymes have been associated with regulation of the cGAS‐STING signaling pathway. Seo et al. revealed that tripartite‐motif containing (TRIM) E3 ligase TRIM56 prompts Lys335 monoubiquitination of cGAS that enhances its dimerization. Moreover, this monoubiquitination is significant for DNA‐binding activity, cGAMP and IFN‐*αβ* production, and anti‐DNA viral immunity.^53^ E3 ligase RING finger (RNF) containing protein RNF185 simplifies the cGAS‐mediated innate immune pathways. During HSV1 infection, RNF185 cooperates with cGAS and reacts with K27‐linked polyubiquitination chains on lysine (K) containing residues K137/384 positions of cGAS, which promotes its enzymatic activity. This catalysis enhances the production of IFNs during viral infections.^50^ In addition, TRIM14 functions as a positive regulator of IFN and targets cGAS. cGAS endures vigorous K48‐linked ubiquitination at lysine (K) 414, which signals the recognition of p62 protein, also called sequestosome 1 (SQSTM1)‐dependent discriminatory autophagic degradation in dormant cells. During infection caused by DNA viruses, TRIM14 recruits protein USP14 to cleave K48‐linked ubiquitin chains of cGAS; therefore, it inhibits interaction with p62‐cGAS and degradation of cGAS.^3^ Additionally, monoubiquitinated cGAS regulation reveals a vital function of RING finger protein that interrelates with C kinase (RINCK) in the cGAS‐mediated innate immunity.^59^

Protein glutamylation is a type of ATP‐dependent PTM that is shown to inhibit virulence factors from regulating bacterial pathogenicity.^60^ Similarly, glutamylation performs an essential role in the regulation of cGAS activity in antiviral immunity.^54^ Glutamylation of cGAS at Glu272 by the tubulin tyrosine ligase‐like (TTLL) enzymatic protein TTLL6 impedes its DNA‐binding capacity, and glutamylation at Glu302 by TTLL4 blocks its fabrication response. This inhibition decreases cGAMP synthesis and obstructs the induction of IFNs upon DNA stimulation in HSV1 infection. Glutamylation is subsequently restored by carboxypeptidases CCP5 and CCP6, which activate transcription factor IRF3 and IFN induction. Additionally, deficiency in CCP5 or CCP6 results in increased susceptibility to DNA viruses.^61^

Ubiquitin ligase Trim38 targets cGAS for SUMOylation during the initial phase of viral contagion. cGAS SUMOylation averts K48‐linked polyubiquitination and cleavage. At an advanced disease stage, Senp2 deSUMOylates cGAS and subsequently degrades through proteasomal and chaperone‐mediated autophagy signaling pathways.^1^ The conjunction of small ubiquitin‐like modifier (SUMO) in cGAS on K335, K372, and K382 sites suppresses DNA binding, nucleotidyltransferase activity, and oligomerization. Conversely, sentrin/SUMO‐specific protease 7 (SENP7) reverses this inhibitory effect by catalyzing the cGAS deSUMOylation during HSV1 infection.^55^

Beclin‐1 autophagy protein functions with the cGAS NTase domain during DNA binding via its CCD domain, and suppresses cGAMP synthesis, impeding IFN production during HSV1 infection. The interaction augments autophagy‐mediated degradation of pathogenic DNA in the cytosolic environment to avoid accidental triggering of cGAS and persistent immune function. Also, beclin‐1 discharges Rubicon, which is a negative autophagy regulator, and triggers phosphatidylinositol 3‐kinase class III responses, and thus induces autophagy to eliminate infectious DNA in the cytosol.^56^ Moreover, cGAMP is also regulated by degradation with phosphodiesterase (PDE) ENPP1.^62^ Recently, poxvirus immune nucleases (poxins) were identified as a family of 2′,3′‐cGAMP‐degrading enzymes. Poxins cleave 2′,3′‐cGAMP to limit STING‐dependent signaling, while removal of the poxin gene (*B2R*) mitigates in vivo vaccinia virus replication.^63^

Microbial inflammation is mediated by the activation of inflammatory caspases (caspase‐1, and caspase‐4/5 in human, or caspase‐11 in mouse). Hence, a balance between IFN production and inflammasome activation is essential for immune homeostasis.^64^ In canonical and noncanonical inflammasome initiation, caspase‐1 cleaves cGAS at Asp140/157 in DNA virus infections, and dampens cGAS‐STING‐mediated IFN production.^57^

#### 4.1.1 Regulation of the STING‐TBK1‐IRF3 Immune Pathway

Regulation of the STING‐TBK1‐IRF3 immune cascade is essential for an antiviral immune response,^9^ which is tightly regulated by ubiquitination and phosphorylation. TRIM56 and TRIM32 ubiquitin ligases bind to STING, mediate K63‐linked ubiquitination of STING, and assist in STING dimerization, as well as interact with TBK1. TRIM32 is significant for the STING‐TBK1 interface following Sendai virus (SeV) or HSV1 infectivity.^11^ Ubiquitin ligase RNF5‐mediated K48‐linked ubiquitination negatively regulates STING and degrades upon viral infection.^65^ RNF26 is recognized as an E3 ligase for K11‐linked polyubiquitination of STING at the equivalent Lys150 STING residue. Likewise, RNF26 also negatively regulates STING in innate immune signaling.^66^ STING is also phosphorylated by ULK1 kinase following DNA or cGAMP stimulation resulting in reduced IRF3 stimulation in a negative‐feedback loop to regulate STING activation.^67^ Conversely, TBK1 phosphorylates STING and positively regulates STING signaling instead.^68^ Mukai et al. showed that palmitoylation inhibitor 2‐bromopalmitate (2‐BP) subjugates palmitoylation of STING and diminishes IFN response; hence, palmitoylation of STING at the Golgi is vital for STING activation.^69^ Franz et al. confirmed that it is not obligatory for STING to prompt IFN induction in RNA virus infection, but also discovered that STING is essential to limit the replication of several RNA viruses.^70^ Zhang et al. reported that nucleotide‐binding leucine‐rich repeat comprising protein NLRC3 prevents appropriate trafficking of STING, and reduces STING‐mediated immune activation in reaction to cytosolic DNA, c‐di‐GMP, and DNA viruses. NLRC3 links to STING and TBK1, which hinders the STING‐TBK1 association, as well as subsequent IFN production.^71^ Prabakaran et al. recently discovered that DNA sensing prompts the cGAS‐STING immune signaling pathway to trigger TBK1, which phosphorylates IRF3 for IFN expression. Additionally, it phosphorylates p62 to degrade STING and decrease the subsequent response.^72^

### 4.2 Evasion of DNA Sensing Pathway by Viral Proteins

Cellular recognition of infectious nucleic acids is necessary for the primary defense mechanism against infectious diseases. Conversely, infections have developed comprehensive escape routes by focusing on host DNA sensors, adaptor proteins, and transcription variables to boost progressive diseases. Comprehension of infection avoidance of the innate immune defenses is still in its early stages and requires extensive elaboration.^73^

#### 4.2.1 cGAS‐Mediated Immune Responses

**Figure 4:**
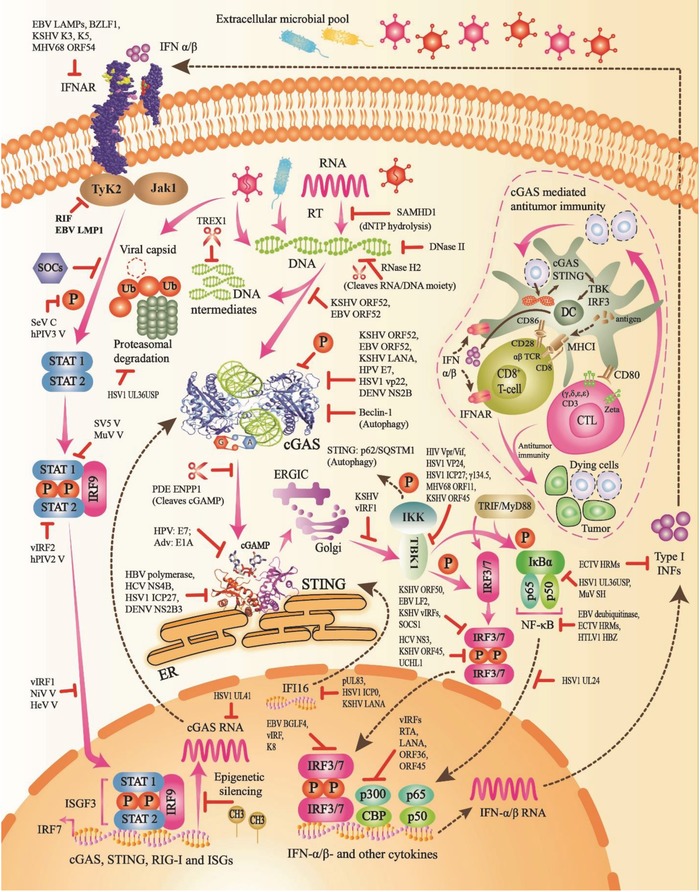
Innate immune regulation of cGAS‐STING‐mediated immune pathways by the host or viral elements. Regulation of cGAS‐mediated immune pathways include i) neutralization of viral nucleic acids, capsid, and proteins by host elements, ii) inhibition of DNA binding to cGAS by viral proteins, iii) inhibition of cGAS activity, cGAS downstream signaling, and its expression by viral‐encoded proteins, phosphorylation, methylation and autophagy, iv) inhibition, blockade, and activity prevention of cGAMP, STING‐TBK1, IRF3/7, NF‐κB, ISGs, IFNs, JAK/STAT signaling pathway, and other cytokines by several viral‐encoded proteins and host elements. Additionally, cGAS is indispensable for cGAS‐STING‐mediated antitumor immunity by superior cross‐presentation of tumor‐related antigens to CD8 T‐cells or CTLs. SOCS, suppressor of cytokine signaling; IFNAR, IFN‐α/β receptor; CTL, cytotoxic T‐cell; CD, cluster of differentiation; Ub, ubiquitin.

Several viruses can evade recognition by cGAS‐STING‐mediated immune pathways (**Figure** **4**).^74^ In viral infections of HSV and Vaccinia virus (VACV), as shown in mice, Mn^2+^ is released from Golgi and mitochondria into the cytosol and induces cGAS‐mediated IFN responses to DNA viruses. Increased cytosolic Mn^2+^ promotes cGAS enzymatic activity and subsequent cGAMP binding affinity to the downstream adaptor STING.^1^ Ding et al. used a genome‐wide clustered regularly interspaced short palindromic repeats CRISPR‐associated protein 9 (CRISPR‐Cas9) method to demonstrate the decline of stromal antigen 2 (STAG2), a constituent of the nuclear cohesin complex. Systematically, STAG2 deficiency triggered spontaneous genomic DNA damage, active IFN‐stimulated gene (ISG) expression, and Janus kinase/signal transducers and activators of transcription (JAK/STAT) signaling via stimulation of the cGAS‐STING signaling pathway, which protected against viral infections, including rotaviruses (RVs).^75^

Numerous viral proteins target cGAS‐mediated immune responses. HSV1 ubiquitin‐specific protease (UL36USP) inhibits ubiquitination of viral capsids, and successive protein degradation over its deubiquitination (DUB) action to seize viral DNA releasing into the cytosol.^76^ KSHV and Epstein‐Barr virus (EBV) ORF52 hinder the activity of cGAS enzyme, linking both DNA and cGAS binding.^1^ Furthermore, KSHV ORF52 inhibits cGAS activity, KSHV latent nuclear antigen 1 (LANA), human papillomavirus (HPV) E7, and phosphorylation.^11^ HSV1 virion host shutoff (Vhs) protein UL41 inhibits cGAS RNA activity. HSV1 tegument protein VP22 inhibits the activity of cGAS enzyme and impedes assembly of IFN and its subsequent antiviral genes.^77^, ^87^ Dengue virus (DENV) NS2B protease cofactor targets cGAS for lysosomal degradation, and subsequently prevents IFN production.^78^ Intriguingly, cGAS expression is epigenetically silenced by DNA methylation in a variety of human tumors, which results in loss of cGAS signaling.^79^ Moreover, Ruiz‐Moreno et al. have recently reported that small interfering RNA (siRNA) silences cGAS and reduces the production of IFN.^80^

Moreover, DNA tumor virus oncogenes, containing E7 from HPV and E1A from adenovirus (Adv), effectively inhibit the cGAS‐STING pathway.^81^ Likewise, STING immune responses are regulated through several viral proteins. STING ubiquitination is inhibited by HBV polymerase.^82^ In human macrophages, IFN expression is inhibited by HSV1 ICP27, which targets the TBK1‐induced STING signalosome.^83^ DENV NS2B3 protease complex cleaves STING, following subversion of innate immune signaling to aid viral replication.^84^ HCV NS4B interrupts STING signaling complexes, and KSHV vIRF1 prevents STING association with TBK1.^11^ Additionally, STING is also regulated by trafficking to ERGIC and degradation through autophagy by Golgi and p62/SQSTM1.^72^ Furthermore, cGAMP‐induced activation of STING requires IFI16 for antiviral defense and is regulated by various viral proteins.^85^ Additionally, IFI16 is inhibited by human cytomegalovirus (HCMV) tegument protein pUL83, which results in immune evasion.^86^ HSV1 ICP0 induces degradation of IFI16, and inhibits IRF3 signaling,^87^ KSHV LANA targets IFI16 for degradation during lytic activation.^88^

TBK1 is a critical antiviral immune constituent, which phosphorylates IRF3/7, induces ISGs, chemokines, and IFN‐α/β, and is regulated by numerous viral proteins for immune evasion.^89^ HIV1 auxiliary proteins, Vpr and Vif, inhibit TBK1 autophosphorylation following obstruction of type I and III IFN stimulation.^90^ VP24, a serine protease of HSV1, abrogates the relationship between TBK1 and IRF3, therefore preventing the induction of IRF3 and IFN production.^91^ HSV1 ICP27 interacts with TBK1 and STING, and results in decreased IRF3 assembly and diminished IFN response.^83^ TBK1 is negatively regulated by HSV γ134.5 protein, which promotes in vivo replication and virus dissemination.^92^ MHV68‐encoded open reading frame (ORF11) immune modulator lessens the association between TBK1 and IRF3 and successively prevents IRF3 stimulation.^93^ KSHV ORF45 protein inhibits TBK1‐dependent IFN expression.^73^

#### 4.2.2 Adaptor Protein‐Mediated Immune Responses

STING, Toll‐IL‐1 (TIR)‐domain‐containing adapter‐inducing IFN‐β (TRIF), and MyD88‐mediated IRF3/7, NF‐κB, and IFNs are inhibited by several viral proteins.[qv: 94b] The proteins include EBV deubiquitinase, Ectromelia virus (ECTV) encoded host‐response modifiers (HRMs), and human T‐cell leukemia virus type 1 (HTLV1) HBZ.^3^

Several viral proteins inhibit phosphorylation of IRF, such as HCV NS3,^95^ KSHV ORF45,^96^ EBV BGLF4,^3^ vIRF,^97^ K8,^98^ and ubiquitin carboxyl‐terminal hydrolase L1 (UCHL1).^99^ Besides, IRFs are also suppressed by KSHV vIRFs, EBV LF2, KSHV ORF50, and the cellular protein suppressor of cytokine signaling (SOCS) 1 prompted by HTLV‐1 Tax.[qv: 94a] HSV1 UL24 binds to NF‐κB subunits p65 and p50 and abrogates nuclear translocation during disease, decreasing NF‐κB activity via the cGAS‐STING response.^100^ Mumps virus (MuV) small hydrophobic protein (SH) decreases NF‐κB stimulation by reducing inhibitor of kappa B (IκB) kinase α (IKKα) and kinase β (IKKβ), and p65 phosphorylation, including nuclear translocation of p65 in diseased cells.^101^ HSV1 UL36USP deubiquitinates IκBα and restricts its degradation, subsequently preventing NF‐κB activation during viral infection.^102^ The p300 and CREB binding proteins (p300‐CBP) transcriptional co‐activating proteins are inhibited by LANA, vIRFs, RTA, ORF36, and ORF45.^98^

#### 4.2.3 JAK/STAT Signaling Immune Responses

IFN‐α/β receptor (IFNAR) induces the Janus family protein kinases (JAKs) tyrosine kinase 2 (Tyk2) and janus kinase 1 (Jak1), and influences tyrosine residue phosphorylation, resulting in STAT1 and STAT2 transcription, leading to the stimulation and development of a heterotrimeric complex comprising IRF‐9 (IFN regulatory factor‐9). 103

The JAK/STAT signaling route is interrupted by several proteins related to numerous infectious viruses. EBV LMP2A and LMP2B proteins mitigate IFN production by targeting IFNARs and decreasing phosphorylation of JAK/STAT1.^104^ EBV BZLF1 protein prompts immune evasion by disrupting the induction of the IFN gamma (IFN‐γ) receptor and inhibiting IFN‐γ‐triggered phosphorylation and nuclear translocation of STAT1 tyrosine.^105^ KSHV K3 and K5 downregulate IFN‐γR1 signal transduction and surface expression. This results in impedance of IFN, and progressive obstruction of IFN‐γ‐mediated phosphorylation and transcriptional activation of STAT1.^106^ MHV68 ORF54 functional deoxyuridine 5′‐triphosphate nucleotidohydrolase (dUTPase) degrades IFNAR1 protein and impedes IFN response, comprising STAT1 phosphorylation.^107^

Moreover, EBV LMP‐1 prevents Tyk2 phosphorylation and impedes IFN‐α‐stimulated nuclear translocation and downstream STAT2 transcription.^108^ RTA and LMP1‐stimulated STAT1 tyrosine phosphorylation are nearly absolute due to NF‐κB‐dependent IFN production.^109^ KSHV RIF protein associates with Jak1, Tyk2, STAT2, and IFNAR subunits and blocks activation of Tyk2 and Jak1; subsequently reduced phosphorylation of STAT1 and STAT2 disrupts nuclear accumulation of ISGF3.^110^ Furthermore, KSHV vIRF1 and vIRF2 impedes IRF‐9, phosphorylates STAT1, and inhibits IFN response.^111^ Additionally, SOCS proteins inhibit JAK/STAT pathway signaling.^112^ SeV C and human parainfluenza virus (HPIV) type 3 V proteins impede STAT phosphorylation and subsequent activation. Simian virus 5 (SV5) and MuV V proteins trigger degradation of STAT1 protein, whereas hPIV2 V protein prompts degradation of STAT2 protein. Nipah virus (NiV) and Hendra virus (HeV) V proteins avert the nuclear accumulation of STAT1 and STAT2 by obstructing IFN signaling.^106^

### 4.3 cGAS‐cGAMP‐STING in Pursuit of Antitumor Immunity

The number of global human deaths attributed to cancer is a rising concern despite substantial developments in cancer therapies during the past decades.^113^ The occurrence of cancer and innate immunity are closely related. T lymphocytes are necessary for tumor immune responses, and they are produced by cross‐priming of tumor‐related antigens. The dendritic cells (DCs) function as a versatile component of the immune system. The antitumor response of cGAS is triggered by tumor DNA in innate immunity, which promotes IFN induction, major histocompatibility (MHC) class I, and a co‐stimulatory cluster of differentiation (CD) molecules, such as CD86 and CD80.^114^ In cytotoxic T lymphocytes (CTL), CD3 T cell co‐receptor activates cytotoxic T‐cell (CD8^+^ naive T‐cells). CD3 protein complex contains γ chain, a δ chain, and twofold ε chains associated with the T‐cell receptor (TCR) and the ζ‐chain (zeta‐chain) to produce activation signals in T lymphocytes. The TCR, ζ‐chain, and CD3 components establish the TCR complex.^115^

The cGAS‐cGAMP‐STING immune pathway plays a pivotal antitumor function. Active immunity is essential in cellular processes, such as cellular senescence, cell death, and DNA damage repair, which are caused by genotoxic stress.^116^ Impaired genomic DNA, as a result of cancer‐causing agents, such as mitochondrial DNA leakage, etoposide, or radiation, 7,12‐dimethylbenz(a)anthracene (DMBA), and cisplatin, has been revealed as a fundamental cause of the cytosolic DNA in cancerous cells, which may trigger cGAS‐STING‐mediated immunity.^42^ The DCs take up DNA fragments, derived from damaged or cancerous cells, and activate the cGAS‐STING pathway. This activation promotes IFN responses in cancerous cells via the cGAS‐STING response, thereby triggering DC maturation.^117^ Mature DCs present tumor‐associated antigens on MHCI and stimulate CD8^+^ T‐cell priming to eradicate cancer cells through the immune system.^116^ Li et al. reported that STING regulator cGAMP retains a robust antitumor response by stimulating STING‐dependent immunity in a mouse model. cGAMP augments innate immunity by initiating assembly of cytokines and inducing DC production, which results in the cross‐priming of CD8^+^ T‐cells. They concluded that cGAMP is an innovative antitumor molecule and plays a prospective role in anticancer immunotherapy.^118^

### 4.4 cGAS‐STING Pathway Activation by Trafficking and Sensing Cyclic Dinucleotides

The CDNs are significant second messenger molecules in various organisms, including eukaryotes and prokaryotes. Cytosolic CDNs are sensed by STING and prime host cells by activating innate immune responses via IFN.^119^ Likewise, extracellular microbes and dying cells can discharge CDNs. However, the detection of extracellular CDNs (eCDNs) by mammalian cells remains obscure. Numerous bacteria yield CDNs, for example, cyclic di‐AMP or cyclic di‐GMP, as signaling particles. When CDNs interact with the cytosol, they are recognized by STING, which prompts the induction of IFN‐β and various cytokines and chemokines.^119^ CDN interaction with STING incites its relocation from the ER to produce perinuclear punctate assemblies. This trafficking within the cell is disrupted by inactive rhomboid 2 and translocon‐associated β proteins (iRhom2/TRAPβ). Additionally, iRhom2 protein assembles the deubiquitinase eukaryotic translation initiation factor 3S5 (EIF3S5), which acts to promote STING during this process.^117^ STING relocation from the ER and interaction with TBK1 in the perinuclear area, prompts the activation of IRF3 through phosphorylation.^120^ TBK1 phosphorylates the tail domain of the carboxy‐terminal from STING, resulting in the assembly of IRF3 and its subsequent phosphorylation.^36^

A current report demonstrated that cGAS advances the STING functionality via extracellular bacterial CDNs. Also, macrophages from human and murine sources can pick up CDNs by clathrin‐subordinate endocytosis in response to these PAMPs by delivering IFN‐β. Endocytosis ensures the incorporation of eCDNs. Assimilated CDNs openly interact with cGAS, prompting the resultant dimerization, and the establishment of a cGAS‐STING assembly that might initiate downstream signaling. The immune responses to extracellular CDNs are expected at ten‐ to 100‐fold higher concentrations of CDN than those utilized for direct transport into the cytosol by digitonin‐interceded membrane permeabilization, which might suggest why an innate immune response to extracellular CDNs has not been witnessed earlier.^119^ Similarly, cGAS encourages the recognition of CDNs trafficked intracellularly through endocytosis, perinuclear amassing, and consequent STING‐mediated induction of IFN. Therefore, eCDNs include bacterial and damage‐associated molecular patterns (DAMPs) that add to host‐associated microbial crosstalk during health and disease.^119^

Due to the antitumoral impacts of cGAMP, various forms of CDN‐centered STING agonists are, as of now, under scrutiny at clinical preliminaries for several tumor types.^121^ Ritchie *et al*. recently determined that cGAMP could perform a unique function as an extracellular immunotransmitter, to serve as a solvent that is delivered and emitted by tumor cells. They led genome‐wide screens employing the CRISPR system and identified human solute carrier family 19 member 1 (SLC19A1) as the primary carrier of cGAMP and numerous CDNs, comprising the new drug 2030‐bisphosphosphothioate‐cyclic‐di‐AMP (2030‐CDAS). These findings would provide further understanding regarding cGAMP's function as an immunotransmitter and help in the advancement of the added focus on CDN‐centered cancer therapy.^121^

### 4.5 cGAS‐STING and Antitumor Activity of Cyclic Dinucleotides

In the instigation of antitumor activity, the deployment of noncanonical cGAMP has triggered the synthesis of various noncanonical CDN analogs. ML RR‐S2 CDA shows enhanced in vitro and in vivo anticancer prospects, and activation of STING.^122^ Moreover, cGAMP combinatorial therapy and 5‐fluorouracil (5‐FU), a DNA disrupting chemotherapeutic drug, displayed potent antitumor activity. Additionally, exogenous radiation therapy and treatment of cGAMP reciprocally amplify antitumor activity.^123^ This radiation and cGAMP immune therapy^124^ motivated investigators to enhance therapy outcomes of radiation and synthetic CDN combinatorial therapy. CT‐guided radiotherapy (RT), in combination with R_P_ (R_P_ dithio CDN molecules), shows synergistic anticancer potential in localized and advanced tumors in a pancreatic cancerous mouse model.^125^

Hypoxic tumors successfully evade immunological stress and antitumor responses by various mechanisms. Wu et al. revealed that microRNA (MiR)‐93, miR‐25, and hypoxia‐responsive miRNAs significantly downregulate cGAS expression in the immunosuppressive tumor microenvironment, thereby improving cGAS DNA sensing expression outcomes in an antitumor immune response.^126^

## 5 Targeting Innate Immune Agents for Immunotherapy

Nucleic acid sensing by innate receptors triggers immune defenses against invading pathogens via the release of IFNs induced by ISGs. Similarly, ISG signatures traced in autoinflammatory and autoimmune conditions involve the contribution of nucleic acid‐sensing pathways.^127^ Immune evasion strategies of malignant cancers lead to the failure of cancer therapies. However, tolerant innate immunity is activated to counter tumor‐induced immunosuppression as a novel immunotherapeutic strategy for cancer patients. Innate immune targets include cytosolic nucleic acid sensors, including RLRs, non‐RLRs, and various DNA sensors, including cGAS. Further, these pathways can be targeted for potential immunotherapeutic strategies (**Table** **2**).^128^

**Table 2:** Cytosolic nucleic acid sensors and immunotherapy

Sensor	Recognized pathogens	Activation/Recognizing legend	Biological response	Immunotherapy	Reference
NLRP3	Influenza virus, SeV, adenovirus, *Mycoplasma pneumoniae*	Bacterial RNAs, DAMPs	Interleukin‐1β (IL‐1β)	Targeting tumor microenvironment via inflammasome/IL‐1 blockade	129
PKR	Bacillus subtilis, encephalomyocarditis virus (EMCV), Theiler's murine encephalomyelitis virus (TMEV), Semliki forest virus (SFV)	dsRNA, short 5′‐ppp RNAs, bacterial RNA, i.e., Bacillus subtilis trp 5'‐UTR	IFN	Suppressing nc886/PKR's oncogenic role, PKR phosphorylation of factor‐2 alpha (eIF2α) inhibits HCV, targeting of PKR and PACT for pharmacological PKR inhibition	130
IFIT	Newcastle disease virus, SeV, dengue virus 2 infections (DENV2)	5'ppp viral ssRNA, adenylate uridylate (AU)‐rich viral RNAs	IRF, IFN	IFIT binding with eIF3 suppresses translation initiation complex and inhibits protein translation, regulation of IFIT2 by Wnt/β‐catenin immune signaling in human colorectal carcinogenesis	131
NOD2	Human respiratory syncytial virus, Borrelia burgdorferi, *Bacteroides vulgatus*	Viral ssRNA, muramyl dipeptide (MDP)	IRF, IFN, pro‐inflammatory cytokines	Activation of NOD2 to induce vigorous cell‐based anti‐tumor innate immunity, targeting of NOD2 ligand MDP and SNPs, epicutaneous (EC) immunization of TNP‐Ig and MDP NOD2	132
ZBP1/DAI	*Human cytomegalovirus*, influenza A virus (IAV)	poly(dA‐dT), VACV DNA, *E. coli* DNA, CT DNA, mtDNA	IRF3, IFN	Regulation of ALD‐DNA‐stimulated macrophage M2b polarization in SLE disease	133
LRRFIP1	*Listeria monocytogenes*, HCV, VSV	GC‐rich Z‐form dsDNA, AT‐rich B‐form dsDNA	IRF3, IFN, IFN‐β	High baseline LRRFIP1 induction in glioblastoma multiforme (GBM) is linked with improved activity to teniposide type II topoisomerase inhibitory agent, LRRFIP1 shRNA lentivirus as prevention strategy for Deep vein thrombosis (DVT), LRRFIP1 induces IFN‐β and inhibits HCV infection in hepatocytes, LRRFIP1 silencing backs the epithelial‐mesenchymal transition (EMT) through inhibitory response of Wnt/β‐catenin	134
DDX41	HSV1, pseudorabies virus, swine virus	B‐form DNA poly(dA:dT), Z‐form DNA poly(dG:dC), c‐di‐GMP, dsDNA	IRF3, IFN, IFN‐β	Somatic DDX41 p.R525H mutation in acute myeloid leukemia (AML), cyclic di‐GMP/YSK05 liposome' for cancer immunotherapy, DDX41 as an effective adjuvant for the G‐based DNA vaccine	20, 135
Ku70/80	HSV1, herpes simplex virus‐2 (HSV‐2), modified vaccinia Ankara (MVA), intradermal infection	DNA DSBs	IRF1, IRF7, IFN‐λ1	Ku70 predicts results of RT in prostate cancer, EAF2 as a critical factor mediating androgen protection of DNA damage via Ku70/Ku80 in prostate cancer, Ku70 silences chemo‐sensitizes gemcitabine in pancreatic cancer cells, target therapy for radiosensitization of Glioblastoma multiforme (GBM) with hyper‐activated UBE2S, ku70/80 as prognostic tool to envisage the reaction to chemoradiation in locally progressive rectal cancer (LARC)	136
MRE11	HSV, *Listeria monocytogenes*, adeno‐associated virus (AAV)	dsDNA, MRN complex	IRF3, IFN	MRE11 as a prognostic biomarker for PARP‐inhibitor therapeutic response and MRN complex therapy, MRE11 in DNA repair and autophagy in cancer therapy, inhibition of adeno‐associated virus by MRN complex	137
DNA‐PK	VACV, HSV1	DSB	IRF3, IFN	Regulation of DNA‐PK in asthma therapy, anti‐DPK3‐scFv as a novel biological radiosensitizer for cancer therapy, DNA‐PK_CS_ inhibitory agent KU60648 as a promising radiosensitizing mediator for osteosarcoma	138
AIM2	Coxsackievirus B3 (CVB3),	dsDNA	IL‐1β, IL‐18	AIM2 co‐immunization helps CD8(+) T‐cell production and amends CVB3 stimulated chronic myocarditis, AIM2‐adjuvanted viral capsid protein 1 (VP1) vaccine for CVB3 therapy, AIM‐2 as antigen‐specific active immunotherapy for glioma patients	139
IFI16	HIV‐1, *listeria, Francisella*, EBV, hepatocellular carcinoma	ssDNA, dsDNA	IFN	IFI16 is an exclusive host sensor protein associated in the EBV infection cycle evincing it a prospective therapy to fight EBV‐related infections, IFI16 expression in p16 therapy, Anti‐IFI16 IgG antibodies in infliximab (IFX) therapy, IFI16 in hepatocellular carcinoma (HCC) therapy	26, 140
Pol III	Adenovirus, HSV1, EBV, *Legionella pneumophila*, varicella‐zoster virus (VZV)	B‐form dsDNA,	IFN	Targeting Pol III for IFN‐β therapy	141
Sox2	*Listeria monocytogenes*, *Bartonella*, *Staphylococcus, salmonella*, vaccinia virus	dsDNA	TNF, IL‐6, IL‐1β, proinflammatory cytokines	Targeting Sox2 for T‐cells cancer immunotherapy	142
cGAS	HSV1, VACV	ssDNA, short dsDNA, G‐YSD, oxidized DNA, B‐form DNA	IFN	Measurement of cGAS activity in cancer immunity and targeting cGAS‐STING pathway in cancer immunotherapy, inhibition of dsDNA stimulation of cGAS by antimalarial drugs (AMDs)	1

PACT, protein activator of the IFN‐induced protein kinase; LGR5, leucine‐rich repeat‐containing G‐protein coupled receptor 5; eIF3, eukaryotic initiation factor 3; SNPs, single‐nucleotide polymorphisms; TNP, 2,4,6‐trinitrophenyl; ALD‐DNA, activated lymphocyte derivative DNA; shRNA, short hairpin RNA or small hairpin RNA; SLFN11, Schlafen family member 11; avSG, antiviral stress granules; c‐di‐GMP/YSK05‐Lip, c‐di‐GMP encapsulated within YSK05‐liposomes; EAF2, ELL associated factor 2; UBE2S, ubiquitin‐conjugating enzyme E2 S; PARP, poly ADP ribose polymerase; scFv, single‐chain variable fragment; KU60648, water‐soluble analog of NU7441; CD8, cluster of differentiation 8; p16, tumor suppressor gene.

Several promising agents trigger the receptors in cancer immunotherapy. These agents include monophosphoryl lipid A (MPL) in cervical cancer, Bacillus Calmette‐Guérin (BCG) in bladder tumor, flagellin‐derived CBLB502 in hepatoma, CpG‐containing oligodeoxynucleotides (CpG ODN) in glioblastoma, Imiquimod in breast cancer, 852A in hematologic malignancy, poly(I:C)/poly‐ICLC in multiple cancer types, 5′ ppp‐siRNA for transforming growth factor‐beta (TGF‐β) in pancreatic cancer, transforming growth factor‐beta (HVJ‐E) in prostate cancer and gliomas, poly(I:C) in ovarian cancer and pancreatic cancer, 5′ ppp‐siRNA for B‐cell lymphoma‐2 (Bcl‐2) in melanoma, cGAMP in colon cancer, and c‐di‐GMP and STINGVAX in melanoma.^143^

### 5.1 Targeting the cGAS‐STING Pathway for Cancer Immunotherapy

Disease remedial immunotherapy is one of the fundamental techniques for curing infectious diseases altering immunity and impairing human health, especially regarding cancer patients.^118^ Numerous DNA sensing agents have been known to detect exogenous nucleic acids. Nevertheless, several depend on STING to initiate IFN responses. Immune checkpoint blockade immunotherapy is a promising anticancer strategy. Zhang et al. reported that cylindromatosis tumor suppressor (CYLD) deubiquitinase protein promotes STING signaling by stabilizing the protein STING. Subsequently, its deficiency promotes the K48‐linked polyubiquitination and degradation of STING, mitigating the stimulation of IRF3‐responsive genes post‐HSV1 infection or the transfection of DNA ligands. The research discovered that CYLD is a novel checkpoint in the cGAS‐STING signaling pathway.^144^ Marcus et al. revealed that the transfer of tumor‐derived cGAMP to nontumor cells triggers STING. cGAMP administration prompts STING activation and IFN‐β production in myeloid cells and B cells, but not natural killer (NK) cells. The antitumor response of NK cells is primarily based on the cytosolic DNA sensing pathway, and identifies tumor‐derived cGAMP as a significant factor of tumor immunogenicity with inferences for cancer immunotherapy.^145^

Furthermore, cGAS product cGAMP is a unique antitumor immune agent and has prospective advances in cancer immune therapeutics. It augments immunity by promoting the production of cytokines, including IFN‐β, IFN‐γ, and influencing DC activation, which stimulates cross‐priming of CD8^+^ T‐cells.^118^ Recently, excessive high‐dose radiation (20–30 Gy in 1 fraction) was demonstrated to disrupt tumor immunogenicity by prompting DNA exonuclease Trex1 to obstruct cGAS‐STING pathway induction.^146^ In autoimmunity of Aicardi‐Goutières syndrome, it has been exposed that RU.521 is dynamic and elective in cellular immune functionality of cGAS‐mediated immune signaling and decreases induction of IFN in macrophages in a mouse model. RU.521 can assist as a constituent for the progress of prospective autoimmune disease therapy.^147^ A new investigation discloses that acetylation adds to the regulation of cGAS activity and delivers a potential therapy for handling DNA‐mediated autoimmune diseases.^148^

Stimulation of the STING pathway leads to IFN expansion, which triggers ISGs, and subsequent cell death. Similarly, it results in IFN‐independent cell death through IRF3 interaction with mitochondrial Bcl‐2‐associated X protein (Bax) dependent on caspases 9 and 3. Thus, regulating STING mediated apoptosis signaling pathways could improve the anticancer activity of STING.^149^ STING is essential in the antitumor immune response. Transplanted immunogenic tumors in STING‐deficient mice grew swiftly, and CD8^+^ T‐cell priming for tumors was compromised in STING deficient mice, and not with deficient TLRs, MyD88, or MAVS, signifying the vital function of STING pathways in controlling tumor progress.^116^ STING is also critical for antitumor activity during anti‐CD47 handling and for generating adaptive antitumor immunity. Cytosolic cGAS‐STING pathways are activated in DCs with the production of IFNs posttreatment with radiation or CD47 antibody.^150^ The classical IL‐4/IL‐13 signaling and STING mediated antiviral innate immune responses include STAT6. Anomalies in STAT6‐mediated signaling are related to advanced asthma and immune diseases, comprising multiple types of cancer. Hence, targeting STAT6 is a promising therapy for treating related conditions.^151^

## 6 The cGAS‐STING Pathway for Tumorigenesis and Immunotherapy Regulation

The adaptive antitumor immunity is exceptionally reliant on innate immune responses to detect non‐self‐material by PRRs.^152^ Tumorigenesis generally relates to the development of cytosolic chromatin particles and micronuclei, expanding the likelihood of DNA release in an existing cell or cancerous cell‐inferred DNA uptake by DC.^153^ Instigation by the cGAS‐STING pathway invigorates IFN induction in diseased cells or DCs, initiating innate immune responses for anticancer immunity. IFN is an adaptable immune agent identified through cell senescence and inflammation immune response. It is confirmed that IFN immune response is fundamental to the cross‐priming of tumor‐explicit T‐cells.^154^

Currently, significant endeavors have been undertaken to locate a suitable cGAS‐STING agonist for anticancer drug advancement. The cGAS‐STING agonists incite diseased cell senescence and improve adaptive anti‐cancer resistance that might synergize with immunotherapies.^153^ Consequently, it is noteworthy to comprehend the advances of cGAS‐STING focusing on procedures with different immunotherapies, for example, RT, cancer vaccines, immune checkpoint inhibitors (ICI), therapeutic oncolytic virus (e.g., Talimogene laherparepvec (T‐VEC)) therapy in melanoma for enhanced expression of STING),^155^ chimeric antigen receptor T‐cell (CAR‐T) therapy employing single‐chain variable fragment (scFv), CAR‐modified T‐cell delivery through bioactive vehicles, and the use of combinatorial therapy by STING agonist cyclic di‐GMP (cdGMP) for tumor exclusion.^156^

### 6.1 Critical Roles of the cGAS Immune Pathway in Antitumor Effects of Immune Checkpoint Blockade

cGAS is vital for definite immune regulation. Several notable innovations in the last decades have propelled the success of antibody development employing powerful antibody engineering techniques.^157^ Immune checkpoint blockade for tumors depicts the use of antibody therapies that intrude on negative administrative checkpoints and discharge earlier antitumor immune responses. Antibodies concentrating on the checkpoint agents, for example, cytotoxic T lymphocyte antigen 4 (CTLA4), programmed cell death 1 (PD1), and death‐ligand 1 (PD‐L1), have had early accomplishment in the clinics, nevertheless, clinicians have yet to isolate effective techniques used on previous patients, in order to move forward with this treatment method. Henceforth, it inspired further interest into the molecular methodologies of tumor‐characteristic resistance from immune checkpoint blockade, inciting the disclosure of biological systems important to antitumor immunity as defined IFN signaling and antigen presentation.^158^

Significant research displayed that PD‐L1 immune checkpoint blockade reduced antitumor immune responses in cGAS‐deficient mice, implying that cGAS is fundamental for antitumor innate immunity.^159^ In another investigation, Wang et al. indicated that cGAS is essential for the antitumor impact of immune checkpoint blockade in mice. They saw that wild‐type, however not cGAS‐devoid, mice displayed slower development of B16 melanomas in light of PD‐L1 counteracting antibody therapy. Reliably, intramuscular conveyance of cGAMP hindered melanoma development and delayed the endurance of the tumor‐harboring mice. The blend of cGAMP and PD‐L1 antibody applied more grounded antitumor impacts than did either approach alone. cGAMP therapy stimulated DCs and upgraded cross‐presentation of tumor‐related antigens to CD8^+^ T‐cells. These outcomes show that initiation of the cGAS pathway is essential for fundamental antitumor immunity and that cGAMP might be utilized straightforwardly for cancer immunotherapy.^160^

Moreover, immune checkpoint pathways enable tumor cells to escape host immunity. Cancerous cells inducing the checkpoint agent PD‐L1 repress T‐cell activity through interaction with the PD‐1 receptor.^161^ CTLA4^+^‐inducing CD8^+^ T‐cells likewise add to immunological resistance via tumors.^162^ Immune checkpoint blockade drugs, involving anti‐PD‐1, anti‐PD‐L1, and anti‐CTLA‐4 antibodies, can release antitumor immune responses and result in further tumor loss. In any case, immune checkpoint blockade is ineffective in “cold” cancer diseases that are ineffectively penetrated by the immune cells. Immune checkpoint‐related immune pathways of the PD‐1/PD‐L1 axis are essential key players in the regulation of tumor evasion. Though IFN‐dependent upregulation of PD‐L1 is generally investigated, ongoing examination indicated the noteworthy signaling of DNA damage in regulating PD‐L1 induction succeeding RT. The DNA damage‐based expression of PD‐L1 is upregulated by kinase functions of ataxia telangiectasia mutated (ATM), rad3‐related kinase (ATR), checkpoint kinase 1 (Chk1) and cGAS‐STING‐based innate immune pathways, demonstrating the function of signaling DNA damage in PD‐L1‐incited induction. Anti‐PD‐1 and anti‐PD‐L1 antibodies as checkpoint blockade immunotherapies combined with RT were shown to extensively advance the coordinated response ratios in different essential and metastatic cancer therapeutics.^163^ Similarly, current examinations anticipate that binary pathways, i.e., mutational loads of IFN‐γ pathways and DNA damage signaling pathways, are associated with immune regulation of PD‐L1 induction in tumors. Immuno‐radiotherapy is profoundly encouraging, especially for nonresponders to inhibitors of the PD‐L1/PD‐1 pathway. Presentation of new radiotherapeutic advances, for example, heavy‐ion particle or proton treatment, may additionally improve the impacts of immunotherapy.^163^

In contrast, combinatorial cancer treatment with STING agonists appeared to improve the impacts of immune checkpoint blockade. The tumor drug STINGVAX is established by means of the granulocyte‐macrophage colony‐stimulating factor (GM‐CSF) with bacterial or assembled CDNs.^164^ Therapy of STINGVAX actuated anti‐tumor immune responses in numerous tumor models.^164^ STINGVAX coupled with ML‐RRS2‐CDA, an objectively structured phosphodiesterase‐resistant c‐di‐AMP (CDA) diastereomer with the phosphate joined linkage as cGAMP, has indicated improved antitumor adequacy contrasted with canonical c‐di‐AMP. Significantly, ML‐RR‐S2‐CDA comprehensively enacts distinctive human STING variations recognized by the 1000 Genomes Project.^153^ STINGVAX additionally upregulates PD‐L1 induction in tumors^164^; co‐treatment of STINGVAX with a PD‐1‐blocking immune response augments antitumor immunity and tumor regression.^162^ Thus, cGAMP increases the antitumor impacts of the PD‐L1 antibody.^160^ Strikingly, STINGVAX can prompt tumor dissemination of CD8^+^ T‐cells in the tumor microenvironment, indicating that it can render tumors “hot.” The cGAS–STING pathway is required for the antitumor impacts of immune checkpoint blockade.^160^

Moreover, the instigation of STING‐based innate immune signaling is seen due to DNA damage in tumor‐associated cells.^165^ Signaling drives checkpoint capture of the cell cycle with resultant DNA damage.^166^ Arrest of the G2/M checkpoint is fundamentally critical to avoid cells with DSB reaching mitosis and propagating inaccuracies of mis‐segregation. The collapse of G2/M checkpoint arrest prompts cell cycle advancement into mitosis, along with DSBs, and the consequent arrangement of micronuclei. An ongoing investigation exhibited that micronuclei initiate inflammatory signaling via the detection of the cGAS/STING pathway.^167^ Strikingly, impairment of the STING pathway counteracted the relapse of abscopal tumors once irradiation (IR) and ICIs were consolidated in in vivo mouse models.^167^ The aforementioned discoveries represent a unique pathway where micronuclei are perceived by cGAS‐STING as a fundamental origin of immunostimulation.^42^ ATM actuates STING through the p53‐IFI16 and TNF receptor associated factor 6 (TRAF6) signaling pathways, which transform STING to IRF3‐NFκB‐dependent transcriptional actuation in a cGAS‐self‐sufficient approach.^168^

### 6.2 cGAS‐STING in Tumor Initiation and Metastasis

Cancer immunotherapeutics must accomplish an appropriate balance between powerful antitumor reactions and avoiding inflammation‐intervened tumor development. Being a basic inducer of IFN reactions, it is not unexpected that cGAS–STING can similarly advance tumor inception and development in a phase‐oriented way. In the prostate tumor, intracellular cytosolic dsDNA aggregation increased through the nonmalignant phase to hyperplasia, to phase II, and afterward, decreased in phase III.^169^ STING deficit is associated with tumor initiation and advancement in a mouse model.^170^ Non‐inflammatory Lewis lung carcinoma (LLC) mouse model is connected with expanded tumor development by STING activation,^171^ STING‐damaged colorectal cancer and melanoma cells demonstrate expanded vulnerability to viral disease, for example, HPV E7 and adenovirus E1A.^81^ Similarly, chronic *Helicobacter pylori* disease in gastric cancer brings about aberrant STING activation and downstream IFN signaling in vivo, which is related to tumor size, movement, and metastasis.^172^ Current investigations additionally recommend that STING can obstruct the antitumor immune responses employing numerous regulatory frameworks, for example, expanded regulatory T‐cell access, IL‐10^173^ and IL‐22BP emission, and tumor immune escape by indoleamine 2,3‐dioxygenase (IDO) protein with decreased T‐cell expansion.^174^

The cGAS–STING pathway performs an essential function in the mechanism of tumor metastasis. Specifically, the proteins connexin 43 and protocadherin 7 permit the exchange of cGAMP via gap intersections between tumor cells and astrocytes, inducing IFN and NF‐κB signaling and consequently advancing brain metastasis.^175^ A study involving cGAS knockdown in cancerous cells brought about decreased phosphorylated IRF3 and IFN in co‐cultured astrocytes and is related to diminished metastasis in the brain.^175^

In a different study, Demaria et al. showed that the intratumoral administration of cGAMP in lung metastasis in mice postponed the development of contralateral tumors.^176^ As it has been observed that, cGAS–STING signaling can deliver a paracrine impact on tumor metastasis; however, further examination is justified to decide on tissue specificity of this impact and for clinical benefit.^177^ Similarly, vascular endothelial growth factor receptor 2 (VEGFR2)^+^CD31^+^ tumor endothelium cells in a melanoma model of B16 mouse created raised ratios of IFN‐β when exposed to cGAMP or tumor DNA, through endothelial determined IFN generation prior to lymphocyte invasion into the tumor region. cGAMP transfer across gap junctions features both the success of cGAS–STING‐mediated innate immunity and possible adverse effects of cGAS–STING‐based treatment.^176^

Harlin et al. declared a firm connection amid tumor penetrating CD8^+^ T‐cells and the induction of chemokine in metastatic melanomas. In a subcategory of melanoma metastasis, it was recommended that decreased primary expression of chemokines is a critical factor in restricting active T‐cell relocation and, accordingly, a viable antitumor immune function.^178^ cGAS‐STING‐mediated innate immune pathway IFN responses advance tumor metastasis over cytokine‐mediated development of a tolerant premetastatic function, such as through the epithelial‐to‐mesenchymal shift.^179^ The developing features for cGAS–STING‐mediated tumor initiation, development, and metastasis advancement in vitro and in vivo require additional investigation in a clinical background. Likewise, through any immunotherapy, regulating the cGAS–STING immune pathway for therapeutic use depends on initiating a robust antitumor immune response, yet limiting tumor‐advancement.^180^

### 6.3 Immune Regulation in Senescence and Tumorigenesis

Cellular senescence is vital to regulate tissue homeostasis, and its cellular disturbance leads to malignancy, premature aging, and age‐related ailments. Cellular senescence is characterized by the growth arrest of injured or aged cells.^41^ Senescence features enlarged and flattened cell morphology, amplified senescence‐related β‐galactosidase (SA‐β‐Gal) response, and alteration in chromatin variation, known as senescence‐associated heterochromatin foci (SAHF).^181^ Even though the DNA damage response (DDR) is connected to senescence activity, the central system is unclear.^12^

The DDR is the main event that leads to senescence and described by the senescence‐associated secretory phenotype (SASP), which comprises induction of inflammatory cytokines, chemokines, extracellular matrix proteins, and growth factors. In addition, several transcription and epigenetic factors, including NF‐κB, bromodomain‐containing protein 4 (BRD4), lysine methyltransferase mixed‐lineage leukemia 1 (MLL1), CCAAT/enhancer‐binding protein β (C/EBP‐β), G9A, p38 mitogen‐activated protein kinase (MAPK), and GATA4, are involved in the upregulation of SASP‐genes.^182^ SASP contributes to several natural courses, such as wound cure, tissue repair, tumorigenesis, or in vivo reprogramming. Therefore, comprehending the regulation of the SASP is vital.^41^

**Figure 5:**
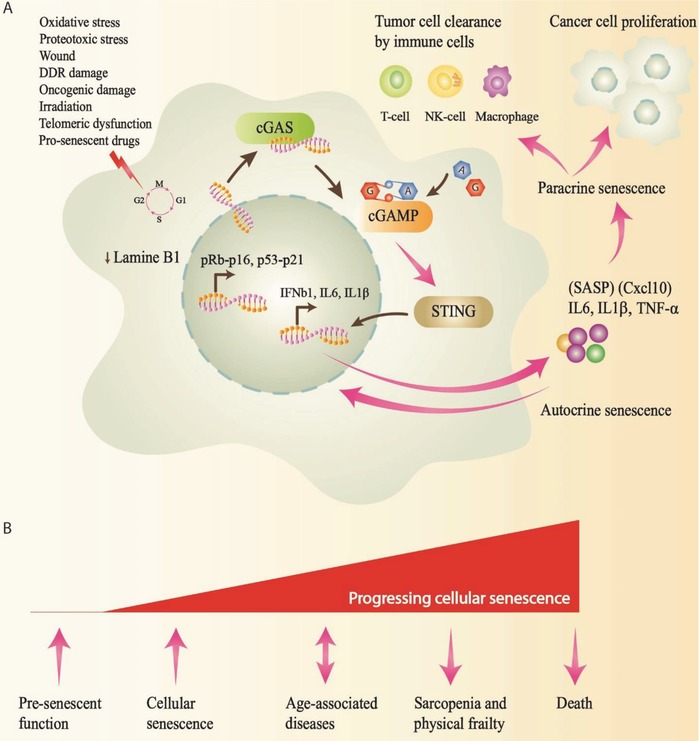
cGAS is essential in cellular senescence and SASP regulation. A) Senescence is triggered by various cellular stresses and cell damage, succeeding the accrual of cytosolic DNA. Consequently, cGAS recognizes DNA and triggers the cGAS–STING pathway to produce SASP factors and induce autocrine and paracrine senescence. Anti‐inflammatory cytokines mediate the clearance of tumor cells by immune cells, whereas pro‐inflammatory cytokines enhance tumorigenesis. B) The processes that lead to cellular senescence, age‐associated diseases, and fundamental aging mechanisms. Interacting with these processes may provide possible therapeutic measures to improve human health.

At the molecular level, the p53‐p21^WAF1^ and pRb‐p16^INK4a^ tumor suppressor pathways regulate the implementation and preservation of senescence. Additionally, SASP components, including IL‐6, IL‐8, and chemokine interferon‐γ inducible protein 10 kDa (CXCL10), support growth arrest in the adjacent cell and eliminate senescent cells.^12^ DNA damage primes the accumulation of cytosolic DNA and activates the cGAS‐STING pathway. Interestingly, DNA damage results in IFN production, which amplifies the DDR and induces cellular senescence.^183^ Endogenous DNA sensing is an essential regulator of senescence and the SASP in the cGAS‐STING pathway. Senescent cells involved in the cGAS‐STING pathway regulate the SASP and assist autocrine and paracrine senescence. Furthermore, activation of cGAS centers on the degradation of the nuclear membrane constituent lamin B1, and the presence of CCFs in senescent cells (**Figure** **5**A).^184^

Also, inducers of cellular senescence include oxidative stress, proteotoxic stress, wounds, DDR damage, oncogenic damage, irradiation, and telomeric dysfunction. The pro‐senescent drug is based upon cGAS‐STING signaling to initiate the assembly of inflammatory SASP components, as shown in Figure 5A.^41^ Recently, Yang et al. showed that cGAS accelerates the spontaneous immortalization of mouse embryonic fibroblasts (MEFs). cGAS deletion retracts SASP, prompted by spontaneous immortalization or DNA detrimental agents, comprising radiation and etoposide. Bioinformatics studies of cGAS expression in human cancer patients display that reduced activation of cGAS is intensely associated with reduced endurance of lung adenocarcinoma patients.^12^ Senescence is a risk factor for most of the chronic cancers and age‐related frailty syndromes, including stresses and sarcopenia in old age (Figure 5B). Moreover, cellular senescence is a potent anticancer strategy, and eradicating senescent cells can defer age‐related dysfunction.^185^ A new study showed that the receptor tyrosine kinase HER2 (also called ErbB‐2 or Neu) potently inhibits cGAS‐STING, thereby disrupting signaling through akt1 recruitment, and prevents the production of cytokines by cancer cells, while embracing senescence and entering apoptosis.^186^

Senescence has risen as a therapeutic focus of high intrigue. The powerful tumor suppressive impacts of senescence have been a research focus for many years, and novel strategies are being sought to treat various cancers. Senescence treatments might be applicable for a variety of age‐related pathologies, such as inflammation, cellular senescence, and cancer.^187^

### 6.4 cGAS Regulates DNA Repair and Tumorigenesis

Molecular transformative investigation of cGAS shows that it has roles supplementary to cytosolic DNA recognition.^188^ Precise repair of DNA DSBs by homologous recombination (HR) maintains genome stability and restrains advancement to tumorigenesis. Detection of severed micronuclei by engaging cGAS links genome vulnerability to innate immunity. However, the prospective contribution of cGAS in DNA repair remains obscure.^167^

cGAS hinders HR along these lines by advancing genome instability, related micronuclear yield, and mitotic destruction. cGAS‐induced hindrance of HR requires its DNA interaction and oligomerization; however, not its synergist action or the downstream innate immune signaling occurrences. By mechanical means, cGAS obstructs RAD51‐induced DNA strand intrusion, a fundamental advance in HR. These outcomes reveal additional cGAS functions, which could be used to understand its contribution to diseases related to genome instability.^189^ In another study, cGAS has recently been shown to associate genomic instability with the innate immune response.^190^ Lately, it is uncovered in mouse and human models that cGAS hinders HR. The ensuing DNA damage incites molecular relocation of cGAS in a manner reliant on importin‐α, and the consequent phosphorylation of cGAS at the site of tyrosine 215 induced by B‐lymphoid tyrosine kinase, which encourages the intracellular cytosolic maintenance of cGAS. Similarly, in the nucleus, the recruitment of cGAS to DSBs occurs and communicates with poly [ADP‐ribose] polymerase 1 (PARP‐1) through the interaction with poly (ADP‐ribose).^190^ The cGAS‐PARP1 cooperation blocks development of the PARP1–Timeless assembly, and in this way, represses HR. They further demonstrated that knockdown of cGAS impedes DNA damage and restrains tumor development both in vitro and in vivo. Consequently, molecular cGAS prevents homologous‐recombination‐mediated repair and advances tumor development, and that cGAS, in this manner, speaks to a potential objective for cancer counteractive immunotherapy.^191^

## 7 Conclusions and Future Prospects

The discovery of cellular receptors and nucleic acid sensors to recognize conserved pathogen structures has a momentously advanced understanding of how the cells sense invading pathogens and trigger innate immune responses and cellular immunity. Prompt recognition of PRRs is an essential strategy for robust and efficient innate immunity. Sensing self‐ and non‐self‐DNA is intensely related to the pathogenesis of inflammatory, autoimmune, cancer, and related diseases. Hence, appropriate host protective cytosolic sensing is critical for mounting active immunity to protect the host from diseases.

Current investigations have concentrated on a consideration of the functions of nucleic acid sensors in host defense. Structural and functional analyses of these sensors have elucidated the mechanisms of innate immune recognition of pathogenic signatures.^1^ Sensing these signatures with various sensors activates the cascade of immune responses that result in the induction of NF‐κB, IRFs, and ISGs, resulting in the assembly of IFNs and pro‐inflammatory cytokines. cGAS is a key cytosolic sensor, which recognizes cytosolic, pathogenic, and self‐DNA. Notwithstanding DNA‐containing viruses and retroviruses, cGAS may recognize DNA from an extensive range of prokaryotes, fungi, and parasites.^3^ Since cGAS ties to and is enacted by DNA irrespective of its sequence, cGAS is proficient at identifying any cytosolic DNA. Similarly, cGAS is an inclusive sensor of pathogens containing DNA or involving DNA at specific cellular phases. Hence, cGAS is extremely important against pathogens of global medical importance.

In recognition of tumor viruses, cGAS‐mediated innate immune responses are confounded by proteins from countless viruses. Tumor viruses prevent recognition, block transcription factor induction, disrupt signaling from IFN receptors, and inhibit the responses of antiviral proteins.^73^ Hence, these immune evasion approaches could be employed to explore novel immunotherapeutic strategies. Careful mixes, designs, delivery vehicles, and paths can be established by aiming at the specific patient population.

cGAS‐STING immune responses are essential in intrinsic antitumor immunity. Potential crosstalk of the cGAS‐STING pathway, comprising TBK1‐IRF3 downstream signaling along with other pathways, such as cytosolic RIG‐I, autophagy, TRAF6, and ubiquitin‐proteasome protein degradation, reveals a vital role in the networking and coordination in sensing RNA/DNA virus infections, autoimmunity, and elimination of other life‐threatening diseases through immunity. However, the regulation and mechanism of action of the cGAS‐STING signaling pathway in numerous disorders remains mostly elusive and must be explored for an effective cure. Likewise, the STING pathway plays an indispensable role in the therapeutic efficacy of cancer immunotherapies for a broader immune response. Intriguingly, CDNs function as STING agonists and activate by traversing cell membranes through a recently discovered major transporter‐SLC19A1.^132^ Prospectively, understanding intracellular CDN trafficking for STING activation is significant for improved immunotherapeutic treatment of cancer and inflammatory diseases. Additionally, cGAS plays critical roles in tumor metastasis, antitumor response via immune checkpoint blockade, and DNA repair, which offer enhanced tumor immunosurveillance, combinatorial therapeutics, and hold promise for successful cancer immunotherapy.

Furthermore, targeting senescence inflammatory pathways in age‐related pathologies may be beneficial in extending the human healthspan. Similarly, it is favorable that patients with immune system sickness, malignancy, age‐related ailments, and infections would all be able to benefit from focusing on the cGAS‐cGAMP‐STING pathway. Additional understanding of the proper regulation of DNA sensors and their biological responses in cellular immunity could be a powerful tool for targeting immunotherapy and the primary focus of future cancer research.

## Conflict of Interest

The authors declare no conflict of interest.
